# Mitochondrial Non-Coding RNAs Are Potential Mediators of Mitochondrial Homeostasis

**DOI:** 10.3390/biom12121863

**Published:** 2022-12-13

**Authors:** Weihan Sun, Yijian Lu, Heng Zhang, Jun Zhang, Xinyu Fang, Jianxun Wang, Mengyang Li

**Affiliations:** School of Basic Medical Sciences, Qingdao University, Qingdao 266071, China

**Keywords:** mitochondria, non-coding RNAs, homeostasis, epigenetics

## Abstract

Mitochondria are the energy production center in cells, which regulate aerobic metabolism, calcium balance, gene expression and cell death. Their homeostasis is crucial for cell viability. Although mitochondria own a nucleus-independent and self-replicating genome, most of the proteins, which fulfill mitochondrial functions and mitochondrial quality control, are encoded by the nuclear genome and are imported into mitochondria. Hence, the regulation of mitochondrial protein expression and translocation is considered essential for mitochondrial homeostasis. By means of high-throughput RNA sequencing and bioinformatic analysis, non-coding RNAs localized in mitochondria have been generally identified. They are either generated from the mitochondrial genome or the nuclear genome. The mitochondrial non-coding RNAs can directly interact with mitochondrial DNAs or transcripts to affect gene expression. They can also bind nuclear genome-encoded mitochondrial proteins to regulate their mitochondrial import, protein level and combination. Generally, mitochondrial non-coding RNAs act as regulators for mitochondrial processes including oxidative phosphorylation and metabolism. In this review, we would like to introduce the latest research progressions regarding mitochondrial non-coding RNAs and summarize their identification, biogenesis, translocation, molecular mechanism and function.

## 1. Introduction

The mitochondrion is a eukaryotic organelle. Hitherto, it is widely accepted that mitochondria originated from a class of endosymbiotic aerobic proteobacteria in the ancestral eukaryote, because they have a double-membrane structure and their own nuclei-independent genome, which encodes mitochondrial-specific tRNA, rRNA and some conserved enzymes expressed in archaea. The proteobacterium entered the eukaryote and escaped from digestion. It consumed excess oxygen in the environment and supplied energy to the host cell, eventually evolving into mitochondrion [1]. 

Correspondingly, the fundamental function of mitochondria is being in charge of the aerobic respiration and energy production of the eukaryote. In the oxygen-enriched environment, mitochondria aerobically catabolize glucose and fatty acids through the tricarboxylic acid cycle (TCA) and β-oxidation, respectively. The generated reducing equivalents are utilized to synthesize ATP via oxidative phosphorylation (OXPHOS). Over 90% of ATPs in the human body are supplied by mitochondria, indicating the central role of mitochondria in energy production and catabolism. Mitochondria are also involved in the anabolism of many substances in the body including urea, amino acids, long-chain fatty acids and phospholipids [2]. Besides metabolism, mitochondria also regulate cellular Ca^2+^ homeostasis via a series of molecular machineries localized in the inner membrane, such as the mitochondrial uniporter complex and the mitochondrial permeability transition pore [3]. In addition, mitochondria indirectly affect gene expression in an epigenetic way. The abundance of acetyl-CoAs generated from aerobic catabolism can serve as the substrates for histone acetylation [4]. Thus, the homeostasis of biological processes including aerobic respiration, metabolism, and substance transport is essential for cell viability. To maintain homeostasis, mitochondria need a complex regulatory system to fulfill their versatile roles. Although self-governed genome and protein synthesis machinery have been retained in mitochondria during evolution, most endosymbiont genes have been integrated into the nuclear genome; only tRNAs, rRNAs and 13 proteins involved in the respiratory chain, including mitochondrially encoded NADH dehydrogenase 1, 2, 3 (MT-ND 1, 2, 3, 4 L, 5, 6), cytochrome c oxidase I, II, III (MT-CO 1, 2, 3), ATP synthase 6, 8 (MT-ATP 6, 8) and cytochrome b (MT-CYB) are encoded by the mitochondrial genome [5]. It means that mitochondria need extra factors to govern DNA duplication, gene expression, transport of ions and metabolites, catabolism and anabolism. To date, 1136 proteins have been identified in human mitochondria [6]. Besides the 13 mitochondrially encoded proteins, the rest are all encoded by the nuclear genome and translated by cytoplasmic ribosomes. Therefore, at the molecular level, strict regulation mechanisms on mitochondrial gene expression and protein import are essential for maintaining functional homeostasis of mitochondria. However, although some protein transporting machineries have been identified in the mitochondrial membrane [7], the processes of protein import have not been fully explored. Moreover, the regulation mechanisms of gene expression in mitochondria have not yet been completely understood.

Non-coding RNAs (ncRNAs) are defined as a class of RNAs which cannot be translated. In a narrow sense, they generally function as post-transcriptional regulators, such as microRNAs (miRNAs), long non-coding RNAs (lncRNAs), circular RNAs (circRNAs) and piwi-interacting RNAs (piRNAs). They are usually transcribed and retained in the nucleus, or transported into the cytoplasm after being generated [8]. By the development of RNA related biological technology, ncRNAs are also identified in mitochondria (Table 1). These mitochondrial ncRNAs (mt-ncRNAs) are either generated in mitochondria, or transcribed by the nuclear genome and further imported into mitochondria. Their functions and molecular mechanisms have also been gradually revealed, and are involved in gene expression, redox regulation, protein transport and so on. In this review, we would like to introduce the biogenesis, transport, mechanisms and biological roles of the mt-ncRNAs. The described evidence suggests that mt-ncRNAs are necessary for mitochondrial homeostasis. 

## 2. Mitochondrial miRNAs

MiRNAs are a class of endogenous single stranded non-coding RNAs with a length of about 20–25 nucleotides. Ordinarily, the primary transcripts of miRNAs (pri-miRNAs) are synthesized by RNA polymerase II in the nucleus, and further cleaved into precursor miRNAs (pre-miRNAs) with a hairpin structure by DROSHA and DGCR8 proteins. Pre-miRNAs are exported to the cytoplasm by exportin-5 and then processed by RNase III Dicer to generate mature single stranded miRNAs. Functionally, miRNAs negatively regulate gene expression. They associate with proteins including RNases to constitute the RNA-induced silencing complex (RISC) and target the 3′ untranslated region (UTR) of mRNAs, which are complementary with the sequences of miRNAs. As a result, the target mRNAs will be degraded, or translationally inhibited [24]. 

In 2006, Lung et al. initially detected a 23 nt ncRNA named Mt-3, which is derived from the D-loop region of the mitochondrial genome, as well as four nucleus-encoded miRNAs including let7f-1, let-7g, miR-122a/b and miR-101b in mitochondria of the mouse liver and kidneys [25]. With the development of the high-throughput RNA sequencing technique and methods of mitochondria purification, a growing number of miRNAs have been identified in mitochondria of mammalian cell lines or tissues. According to the reported data, almost all the sequences of the mitochondrial miRNAs originated from nuclear genomes, implying the tissue-specific expression profiles and novel import pathways of mitochondrial miRNA [9,10,11,12,13,14,15,16,17,26]. The sequences of three miRNAs including miR-1974, miR-1977 and miR-1978, are matched with those of tRNAs and rRNAs genes in the human mitochondrial genome [12]. Two pre-miRNAs including pre-miR-let7b and pre-miR-302a were also suggested to be generated from the human mitochondrial genome [13]. However, it cannot be excluded that these candidates might be transcribed from the redundant mitochondrial genome-equivalent sequences in the human nuclear genome. The actual presence of mitochondrial genome-derived miRNA is unclear. 

Several proteins have been reported to possibly mediate import of mitochondrial miRNAs. Ago2 is the core of RISC that directly binds mature miRNA. It has RNA cleavage activity and interacts with translation initiation factor EIF6 and the 60S ribosome subunit RPL7A, thus leading to degradation or translation inhibition of the miRNA-targeted mRNAs [27,28]. Nevertheless, Ago2 was also detected in the mitochondria and associated with transcripts of mitochondrial DNA [10,12,14]. Therefore, Ago2 was inferred to transport miRNAs from the cytoplasm into mitochondria. However, there is still no solid evidence to prove it. Another potential transporter of mitochondrial miRNAs is polynucleotide phosphorylase (PNPase). PNPase is an RNA binding protein, which is translocated into the intermembrane space of mitochondria in a novel pathway requiring translocase of outer membrane (TOM), translocase of inner membrane (TIM) and ATP-dependent zinc metalloprotease Yme1. The N-terminal mitochondrial targeting sequence of precursor PNPase was cleaved after passing through TIM, then the mature PNPase was pulled into the intermembrane space through TOM by inner membrane-localized Yme1 and formed trimer [29]. PNPase was reported to regulate the importation of small RNAs including the RNA components ribonuclease P (RMRP) and the mitochondrial RNA-processing (MRP) RNA into the mitochondrial matrix [30]. There is also a study showing that PNPase was involved in mitochondrial miRNA import. Shepherd et al. observed that PNPase facilitated the translocation of miR-378 into mitochondria. They also found that PNPase associated Ago2 in the mitochondria, implying there might be cooperation between them in miR-378 transport [31]. A fraction of PNPase has also been detected in the mitochondrial matrix showing 3′-5′ exoribonuclease activity [32]. However, abolishment of the exoribonuclease activity by mutation does not affect RNA import, suggesting PNPase transports RNAs into mitochondria in an exoribonuclease-independent manner [30]. On the other hand, PNPase might import RNAs into the mitochondrial matrix by cooperating with other proteins, such as TIM. It was suggested that a 0–2 nt of 3′ overhang in target RNA is required for the transporting activity of PNPase [33]. Perhaps miRNA can be imported by PNPase because of its short 3′ overhang. In addition to mature miRNAs, the existence of pre-miRNAs in mitochondria were also detected [13]. Coincidently, the import function of PNPase is specific to target RNAs with stem-loop structures, which pre-miRNAs have [30]. Therefore, it is possible that PNPase was involved in mitochondrial pre-miRNA transport. It can be also inferred from the presence of mitochondrial pre-miRNAs that there might be machinery for pre-miRNA processing in mitochondria. By means of immunoblotting, Wang et al. found that Dicer, which generates the mature form of miRNA, was present in the component of mitochondrial outer membrane and the matrix of the rat hippocampus [16]. However, Das et al. found that Dicer was not present in the mitochondrial fraction of heart tissue [26]. These contradictory results might be caused by differences in methodology. It needs to be further verified whether Dicer mediated the processing of mitochondrial pre-miRNAs.

**Figure 1:**
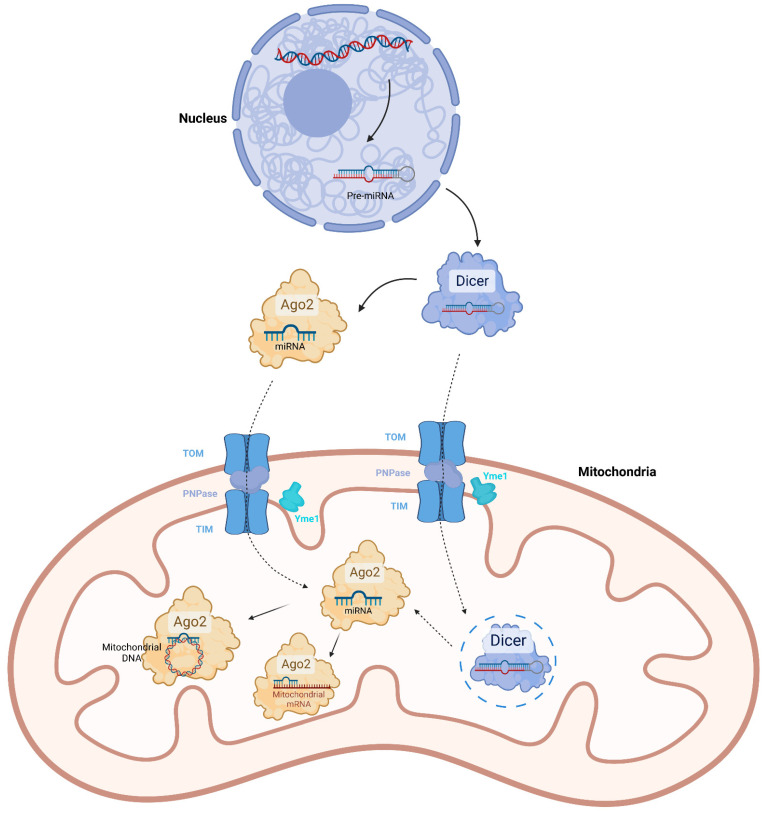
The biogenesis, translocation and function of mitochondrial miRNAs. Mitochondrial miRNAs are transcribed from nuclear genome. The mature miRNAs associated with Ago2 are translocated into mitochondria by PNPase. Pre-miRNAs are also present in mitochondria, which might be further cleaved into mature form by Dicer. However, the mitochondrial localization of Dicer is controversial (The related molecules and processes are labeled in dotted-line). The mitochondrial miRNAs either interact with mRNAs to regulate translation, or interact with DNA to regulate transcription. These mechanisms are Ago2-dependent.

By means of immunoprecipitation assays, it has been demonstrated that mitochondrial miRNAs also function by directly interacting with target mRNAs in an Ago2-dependent manner [15,26,34,35,36]. However, unlike cytosolic miRNAs which are well characterized to negatively regulate gene expression, the molecular effects of mitochondrial miRNAs were controversial; some can promote the translation of target mRNAs without affecting their level. For instance, miR-21 can interact with the ORF of mt-CYTB mRNA and increase the protein level of mt-CYTB [35]. Some other miRNAs, such as miR-181c and let-7a, showed opposite activity. They even affect the expression of the adjacent downstream gene. Das et al. found that transfection of pre-miR-181c mimic into neonatal rat cardiomyocytes could inhibit the translation of mt-COX1, while increasing the mRNA level and protein level of mt-COX2 [26]. However, the in vivo effect of miR-181c being reported by the same research group of Das et al., was inconsistent with the result in vitro; delivery of miR-181c nanovector to the rat through the tail vein could decrease the mRNA level and protein level of mt-COX1 in the heart, as well as those of mt-COX2 [37]. This contradiction might result from the difference between environments in vitro and in vivo, or distinct strategies of forced miR-181c expression. It should be mentioned that the transcription, RNA processing and translation of mitochondrial genome-encoded genes are not compartmentalized by membranes. It means that mitochondrial miRNAs also possibly directly interacted with the polycistronic pre-mRNA. This might affect the splicing of the target mRNA and adjacent mRNA, just like the effect of miR-181c on mt-COX2. However, it needs to be demonstrated by a wide range of hybridization analyses. The mechanisms which determine the positive or negative regulatory activity on gene expression of mitochondrial miRNAs, have not been understood. In addition, miRNAs such as miR-2392, can directly target the mitochondrial DNA sequence and partially inhibit polycistronic DNA transcription [38]. This mechanism is also Ago2-dependent (Figure 1). On the other hand, it cannot be ignored that forced expression of miRNAs in the studies significantly increased the miRNA level in the cytoplasm, which might also indirectly affect the mitochondrial transcription or translation.

## 3. Mitochondrial lncRNA

The linear non-coding RNAs with a length over 200 nt are referred to as lncRNAs. LncRNAs share the same biogenesis process and structure as mRNA. They are transcribed from specific genes. The precursor transcripts of lncRNAs contain exons and introns. The introns will be removed from the pre-lncRNAs through splicing, followed by the addition of 5′ cap and 3′ polyA tail. Thus, the expressions of lncRNAs are also spatio-temporally regulated the same as mRNAs. Although lncRNAs cannot be translated due to lack of ORF, they actually play significant regulatory roles in gene expression. Most lncRNAs are localized in the nucleus and involved in the processes of transcription and splicing. They usually interact with transcription factors or epigenetic mediators, and target gene promoters to regulate transcription. Some of the nuclear-lncRNAs directly interact with DNA templates to inhibit transcription. Others can bind pre-mRNA to affect splicing. There are also cytoplasmic lncRNAs, which function as competing endogenous RNAs or protein scaffolds [39].

**Figure 2:**
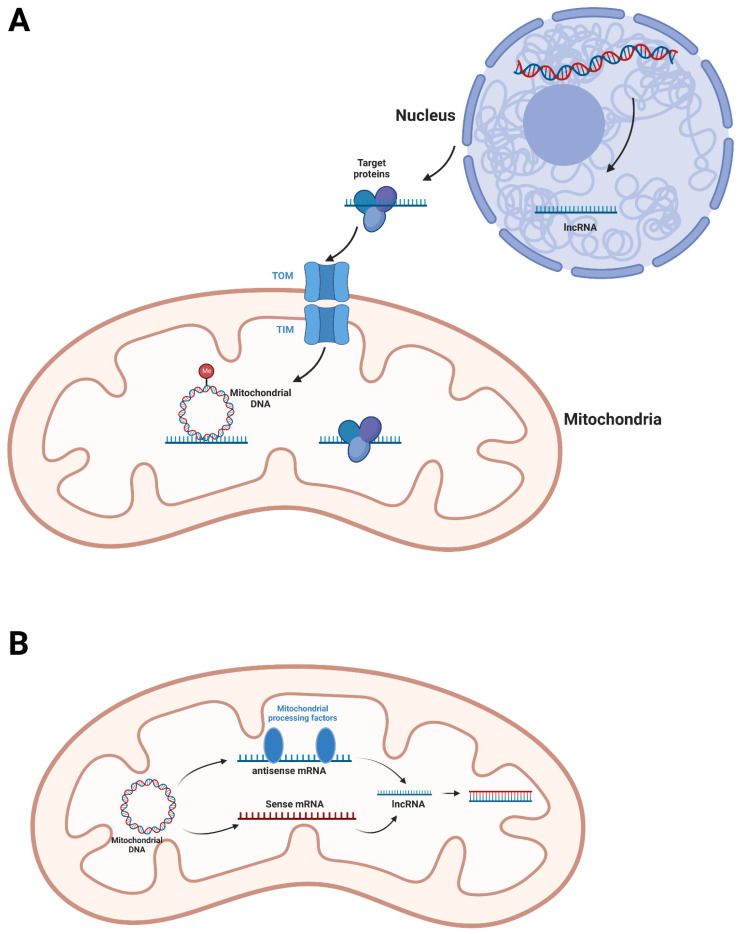
The biogenesis, translocation and function of nuclear genome-encoded mitochondrial lncRNAs (**A**) and mitochondrial genome-encoded lncRNAs (**B**). (**A**) The mitochondrial translocation of nuclear genome-encoded mitochondrial lncRNAs might be correlated with associated nuclear-genome-encoded mitochondrial proteins. These lncRNAs were discovered to function via binding proteins, as well as interact with mitochondrial DNA to affect DNA methylation. (**B**) Mitochondrial genome-encoded lncRNAs might be processed by processing factors of mitochondrial tRNAs, such as MRPP1. They complementally interact with the mRNAs to stabilize the targets.

A fraction of nuclear genomes-encoded lncRNAs were not only present in the cytoplasm or nucleus, but also in mitochondria. For instance, RMRP, which is the RNA component of RNase MRP and essential for mitochondrial DNA replication and RNA processing, has been detected in the mitochondria of mouse cardiomyocytes and myoblasts, as well as HEK293 cells. RMRP was suggested to be imported into mitochondria by PNPase, and retained in the mitochondrial matrix by binding GRSF1 [40]. The RNA component H1 of ribonuclease P, RPPH1, was also detected in mitochondria by in situ hybridization [41,42]. LncRNA SAMMON is distributed in both melanoma cytoplasm and mitochondria. It can interact with p32 and facilitate the mitochondrial localization of p32 [43]. LncRNA GAS5 is enriched in the mitochondria of HEK293T cells and regulates the association of fumarate hydratase (FH), malate dehydrogenase 2 (MDH2) and citrate synthase (CS) by interacting with MDH2 [18]. A lncRNA related to fatty acid β-oxidation (lncFAO) was found to bind HADHB in mitochondria of mouse macrophages and post-transcriptionally affect HADHB level upon stimulation of LPS [44]. It can be concluded from these studies that the above-mentioned nuclear genomes-encoded lncRNAs generally function by directly binding proteins to regulate their translocation, stability, activity or association. Although the mechanisms mediating mitochondrial import of the lncRNAs have not been revealed, some evidence implied that the lncRNA-targeted mitochondrial proteins might be involved in this process. Noh et al. found that overexpression of GRSF1 enhanced mitochondrial localization of RMRP. On the contrary, knockdown of GRSF1 reduced the level of RMRP in mitochondria [40]. Sang et al. observed that exclusion of the MDH2 binding region from the sequence of GAS5 resulted in a loss of mitochondrial localization [18]. Probably, these lncRNAs were imported along with their protein partners through mitochondrial protein translocators. There is also a special case that mitochondrial localization of lncRNA was possibly conditional. Metastasis Associated Lung Adenocarcinoma Transcript 1 (MALAT1) is a well-studied nuclear lncRNA [45]. Zhao et al. found that MALAT1 was also present in the mitochondria of hepatocellular carcinoma cells, while barely detectable in the mitochondria of normal hepatic cells. Mitochondrial MALAT1 also showed mechanisms distinct from other mitochondrial lncRNAs; MALAT1 interacted with mitochondrial DNA and regulated their methylation (Figure 2A). Zhao et al. suggested that mitochondrial translocation of MALAT1 might be mediated by mitochondrial carrier MTCH2 because MALAT1 was demonstrated to interact with MTCH2 by IP [19]. The mitochondrial localization of MALAT1 was also reported by Mohammad and Kowluru. Moreover, they found that mitochondrial translocation of MALAT1 in retinal endothelial cells was induced by high glucose [46]. Maybe metabolic remodeling was involved in the mitochondrial import of MALAT1 in hepatocellular carcinoma cells and retinal endothelial cells.

Actually, the existence of mitochondrial genome-encoded lncRNAs had been verified by Villegas et al. in as early as 2000. They identified a ~1700 nt mitochondrial lncRNA containing the sequence of 16S rRNA and an inverted repeat joined to its 5′ end in mouse sperm and named it SncmtRNA [47]. The 2374 nt equivalent transcript and two antisense transcripts (ASncmtRNA1 and 2) were subsequently identified in human cells by the same research group [48,49]. Although SncmtRNA and two ASncmtRNAs were generated and detectable in mitochondria, they were, nevertheless, concentrated in the nucleus. Whether they function in mitochondria is unknown. By means of RNA sequencing, more mitochondrial genome-encoded lncRNAs were identified. For instance, lncND5, lncND6 and lncCYTB are the antisense non-coding transcripts of ND5, ND6 and CYTB genes, respectively. They were discovered from purified human mitochondria and showed cell-tissue-specific expression. The levels of these three lncRNAs were affected by mitochondrial processing factors such as MRPP1, MRPP3 and PTCD1, which are involved in the processing of mitochondrial tRNAs. This implies that the mitochondrial genome-encoded lncRNAs might be generated from the cleavage of polycistronic mRNAs by processing machineries of mitochondrial tRNAs. Mechanically, all lncND5, lncND6 and lncCYTB could form RNA-RNA duplexes with respective complementary mRNAs, suggesting they might regulate parental gene expression by stabilizing the mRNA [50] (Figure 2B). It is weird that these lncRNAs also showed considerable nuclear localization, such as SncmtRNA and ASncmtRNAs. LncCYTB was especially tracked to aberrantly shuttle into the nucleus in HepG2 cells resembling the above-mentioned MALAT1 [42]. The mechanisms of the lncRNAs shuttling from mitochondria to the nucleus have yet to be revealed. In addition, some potential mitochondrial genome-encoded lncRNAs remain to be fully characterized. MDL1, the lncRNA which is generated from the antisense counterpart of mitochondrial tRNAPro gene and D-loop region, was discovered from the RNA-seq data, as well as its antisense transcript, MDL1AS. The expression, cellular localization and function of MDL1 and MDL1AS have not been studied. They were suggested to be the precursors of some other small RNAs [51]. A lncRNA, named long intergenic noncoding RNA predicting cardiac remodeling (LIPCAR), was identified in the plasma of heart failure patients and was selected as a diagnostic biomarker for coronary heart disease. The sequence of LIPCAR consists of a 5′ partial antisense transcript of the Cytb gene, fused with a 3′ antisense transcript of the COX2 gene [52]. The reason causing the interrupted structure of LIPCAR is unclear. 

## 4. Mitochondrial Piwi-Interacting RNAs

PIWI-interacting RNAs (piRNAs) are ncRNAs of approximately 24–31 nt in length and are found to be enriched in germ cells. They have been established to modulate repression of transposable elements, mRNA stability and translation in germ cells, as well as somatic cells. The biogenesis process of piRNAs is complicated. The longer precursors of piRNAs are generally transcribed from specific genomic regions termed ‘piRNA clusters’. They are further processed into mature piRNAs through two correlated pathways, which are rather conserved across species. Ping-pong amplification is a type of piRNA processing mediated by PIWI proteins, which occurs in ribonucleoprotein granules. PiRNAs in opposite orientation with 10 nt of 5′ complementary overlaps are produced from piRNA precursors in ping-pong amplification, which relies on endonuclease activity of a pair of piRNA-guided PIWI proteins. Phasing is also known as phased piRNA processing. In the process of phasing, PIWI-loaded piRNA precursors are translocated to the mitochondrial outer membrane by RNA helicases, such as Armitage (Armi) and MOV10L1. They are further cleaved by endonuclease Zucchini or PLD6 [53,54]. In addition to Armi/MOV10L1 and Zucchini/PLD6, some other mitochondrial proteins have also been reported to be involved in piRNA processing. For example, BmPAPI, a homolog of TUDOR domain-containing protein PAPI/TDRKH in silkworm was found to recognize symmetrical dimethylarginines in PIWI proteins and interact with them at the surface of mitochondrial outer membrane [55]. Exoribonuclease PNLDC1 binds PAPI/TDRKH on the mitochondrial surface to serve as the pre-piRNA 3′ trimmer in silkworms [56]. What is more is that it was remarkable that a PIWI protein, Piwil1, was observed to localize in mitochondria, raising a possibility of inter-mitochondrial piRNA biogenesis [20]. Consistent with this, a fraction of potential mitochondrial piRNAs were revealed via bioinformatic analysis or RNA-seq. In 2014, Kwon et al. identified 29 piRNA, which might be transcribed from mitochondrial genomes in HeLa cells and HEK293 cells. Data of small RNA-seq showed that they were expressed in mitochondrial subcellular fractions [20]. By means of bioinformatic analysis, Larriba et al. screened out numerous potential piRNAs from sequencing data of small non-coding RNAs in early differentiated germ cells of male mice, gametes of both sexes and in zygotes. The sequences of piRNAs originated from any region in both strands of mouse mitochondrial DNA. Among these candidates, piR-mmu-7,456,245, which showed relatively high expression in all types of cells, was detected to localize in mitochondria [21]. This research group also identified piRNAs by RNA-seq, which might be generated from the mitochondrial genome—or mitochondrial counterparts in the nuclear genome in mouse embryonic gonadal cells—indicating a potential piRNA-mediated communication between mitochondria and the nucleus [22]. Overall, the research on mitochondrial piRNAs is still superficial. Most of the potential mitochondrial piRNAs have not been strictly characterized. Their biogenesis and function remain to be uncovered.

## 5. Mitochondrial circRNA

**Figure 3:**
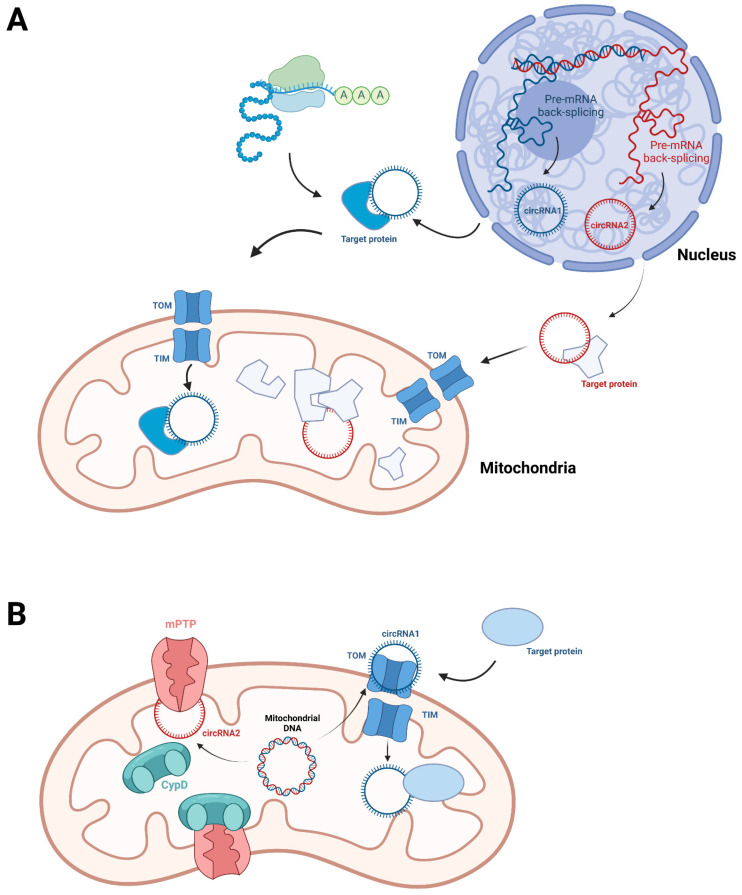
The biogenesis, translocation and function of nuclear genome-encoded mitochondrial circRNAs (**A**) and mitochondrial genome-encoded circRNAs. (**A**) Nuclear genome-encoded mitochondrial circRNAs are generated from back-splicing of pre-mRNA. Their mitochondrial localization might be related to their associated mitochondrial proteins. In mitochondria, these circRNAs interact with proteins to affect their translocation. They also act as protein scaffolds. (**B**) The detailed mechanism of mitochondrial genome-encoded circRNAs biogenesis has not been clear. The mitochondrial genome-encoded circRNAs can interact with proteins to affect their association. For instance, the interaction between CypD and mPTP subunits can be modulated by circRNA. The circRNAs are also found to be co-localized with mitochondrial transport machineries, such as TOM40, to facilitate protein import.

CircRNAs are a class of single-stranded RNAs with a covalently closed circular structure. Most circRNAs are generated from exons of nuclear genome-encoded genes, which are generally flanked by long introns with internal complementary sequences or short interspersed elements (SINE). When they are transcribed into pre-mRNA, the flanking introns will be associated with each other to form a hairpin structure, that promotes the so-called ‘back-splicing’ and biogenesis of exonic circRNAs. Some intronic lariats can escape from debranching to form intronic circRNAs. Most circRNAs are localized in the cytoplasm or nucleus. The cytoplasmic circRNAs generally function as a miRNA sponge, protein sponge, protein scaffold, or even translation template. Nuclear circRNAs are involved in transcription and RNA processing [57,58]. Surprisingly, some nuclear genome-derived circRNAs have also been identified in mitochondria, and shown to interact with mitochondrial proteins and further affect their mitochondrial localization. The results of Zheng et al. indicated circSamd4 is highly expressed in the mitochondria of cardiomyocytes, which interacts with Valosin-containing protein (VCP) and facilitates the mitochondrial localization of VCP [59]. On the other hand, although the mitochondrial translocation mechanisms of circRNAs have not been specially explored, the function of nuclear-derived circSamd4 implies that the nuclear genome-derived circRNAs might be imported into mitochondria with their protein partners, through the machineries for protein translocation. The nuclear genome-derived circRNAs are also reported to act as protein scaffolds. In ESCC cells, circPUM1 is demonstrated to localize in mitochondria and enhance the association between ubiquinol-cytochrome c reductase core proteins 1 and 2 (UQCRC1 and UQCRC2) [60] (Figure 3A). 

As early as 2013, several circRNAs originated from human mitochondrial DNA had been detected by RNA-seq. Hsa_circ_0008882 andshsa_circ_0002363 and were detected in human normal male glans cells hs68 by Jeck et al. [61]. Hsa_circ_0089763, hsa_circ_0089762 and hsa_circ_0089761 were detected in human cell lines including K562, H1hesc, Gm12878 and Sknshra by Salzman et al. [62]. However, their actual existence was not strictly verified at that time. In 2020, Wu et al. reported that 28 circRNAs were highly expressed in the plasma of chronic lymphocytic leukemia (CLL) patients. Intriguingly, the top four enriched circRNAs were all mitochondrial DNA-derived circRNAs including hsa_circ_0089763, hsa_circ_0008882, hsa_circ_0002363 and hsa_circ_0089762, which had previously been detected by Jeck et al. and Salzman et al., respectively. Hsa_circ_0089762 were focally characterized to be localized in the extra-nuclear region and exosomes [63]. Zhao et al. also observed that has_circ_0089762 (also named steatohepatitis-associated circRNA ATP5B regulator, SCAR), hsa_circ_0089763 and hsa_circ_0008882 are abundant in liver fibroblasts from patients with nonalcoholic steatohepatitis (NASH), and further demonstrated their mitochondrial localization [64]. Liu et al. deeply explored the mitochondrial genome-encoded circRNAs in human and murine mitochondria through second-generation sequencing. Totals of 248 and 268 mitochondrial genome-encoded circRNAs were identified in human and murine samples, respectively. Some of them are distributed in both the mitochondria and the cytoplasm. These circRNAs are transcribed from both strands of mitochondrial DNA [23]. Compared with nuclear-derived circRNAs, the circularized regions in mitochondrial DNA lack flanking elements, which facilitate back-splicing. This suggests that the biogenesis machinery of mitochondrial genome-encoded circRNAs may be distinct from that of nuclear-derived circRNAs. Liu et al. found a novel feature that a pair of 4 nt short repeats are, respectively, situated at the positions adjacent to the 5′ junction site and 3′ junction site in approximately 40–50% of these mitochondrial genome-encoded circRNAs, suggesting that it might be related to the biogenesis of circRNAs [23]. The reported functional mitochondrial genome-encoded circRNAs all act by binding protein. The interaction between circRNAs and proteins can disrupt protein association. For instance, SCAR functions by directly binding ATP5B to block the interaction between CypD and the mitochondrial permeability transition pore (mPTP) [64]. In addition, protein import can also be promoted by the association of circRNAs. MecciND1 and MecciND5, two mitochondrial genome-encoded circRNAs, were suggested to promote mitochondrial protein import by Liu et al. [23]. MecciND1 was shown to bind RPA70 and RPA32 while MecciND5 bound hnRNPA1, hnRNPA2B1, and hnRNPA3. Knockdown of MecciND1 and MecciND5 decreased the mitochondrial localization of their protein partners, while it did not alter the total level of protein partners. Moreover, both MecciND1 and MecciND5 can interact with TOM40 and PNPase. TOM40 is a channel-forming protein essential for import of protein into mitochondria. PNPASE is a major mitochondrial RNA importation factor. This infers that MecciND1 and MecciND5 are involved in the translocation of mitochondrial proteins. 

## 6. Mitochondrial ncRNAs Mediate Mitochondrial Homeostasis

Accumulated evidence has demonstrated that mitochondrial ncRNAs regulate OXPHOS, metabolism, mPTP opening, DNA replication and protein translation to maintain the functional homeostasis of mitochondria (Table 2).

Mitochondria provide the vast majority of energy in the cell via OXPHOS, which is modulated by a respiratory chain consisting of Complex I–IV and ATPase. Many mitochondrial miRNAs have been revealed to affect the level of mitochondrial genome-encoded enzymes in the respiratory chain. For instance, miR-378 targets ATP6 mRNA to decrease ATP6 level and ATP production in cardiomyocytes [15,31]. Let-7a post-transcriptionally inhibits ND4 by directly binding its mRNA to alter the activity of Complex I in breast cancer cells [36]. MiR-1 targets ND1 and COX1 mRNA to enhance their translation and ATP production in muscle cells [34]. MiR-21 interacts with the ORF of CYTB mRNA and promotes translation of CYTB to reduce reactive oxygen species (ROS) production [35]. MiR-181c binds to the 3’UTR of COX1 to decrease COX1 protein level, resulting in disorder of mitochondrial respiration and reactive oxygen species generation in neonatal rat ventricular myocytes [26]. Mitochondrial ncRNAs also transcriptionally mediate OXPHOS. MiR-2392 represses the transcription of ND4, CYTB, COX1 and COX2 by targeting mitochondrial DNA to enhance anaerobic respiration in tongue squamous cell carcinoma cells [38]. MALAT1 interacted with multiple loci on mitochondrial DNA, including D-loop, COX2, ND3, and CYTB genes in HepG2 cells. It inhibits the methylation at CpG island 3 of mitochondrial DNA, and promotes the transcription of genes in OXPHOS including ND1-3, 5, 6, COX1, 2 and CYTB, maintaining basal respiration ability and ATP-producing capability [19]. In addition to miRNAs, some mitochondrial circRNAs have also been reported to modulate OXPHOS by directly binding the enzymes lying in the respiratory chain. For example, circPUM1 bonds UQCRC1 and UQCRC2, which are the components of the ubiquinol-cytochrome c oxidoreductase in Complex III [65]. It can maintain the stability of complex III to enhance OXPHOS for ATP production [60]. 

Mitochondria are the center of aerobic catabolism of glucose and fatty acids. All the enzymic machineries in TCA and β-oxidation are encoded by the nuclear genome and translocated into mitochondria. Some mitochondrial ncRNAs are involved in regulation of TCA and β-oxidation by directly targeting these enzymes. FH, MDH2 and CS are tandem enzymes lying in the cascade of converting fumaric acid into citric acid. The acetylation of MDH2 is essential for the formation of FH–MDH2–CS heterotrimer and negatively regulated by SIRT3. GAS5 can recruit SIRT3 to MDH2 and disorganize the complex, resulting in retardation of the TCA [18]. HADHB is one subunit of the mitochondrial trifunctional enzyme. It bears 3-ketoacyl-CoA thiolase activity. LncFAO binds HADHB to increase the protein level and activity of HADHB, promoting fatty acid oxidation in macrophages [44].

MPTP is a nonspecific channel composed of multiple proteins, which spans the inner mitochondrial membrane and outer mitochondrial membrane [66]. MPTP opening is considered as a typical event of mitochondrial dysregulation, including energetic dysfunction, swelling and rupture of organelle, interruption of mitochondrial respiration, releasing of ROS into cytoplasm, loss of inner mitochondrial potential and necrotic cell death [67]. CypD is the regulator of mPTP opening, which binds ATP synthase in mPTP [68,69]. SCAR interacts with ATP5B, which is one subunit of ATP synthase and mPTP regulator [70], to prevent CypD associating with mPTP, and shuts down mPTP. VCP is a transitional endoplasmic reticulum ATPase and is reported to play roles in protein degradation [71,72]. VDAC1 is a channel at the outer mitochondrial membrane and one of the components of mPTP [73]. CircSmad4 can interact with VCP and enhance its mitochondrial import, leading to downregulation of VDAC1 and inhibition of mPTP opening. 

Some mitochondrial ncRNAs are also reported to mediate mitochondrial homeostasis at an integral level. P32 is required for the maturation of mitochondrial 16S rRNA and protein translation in mitochondria [74]. LncRNA SAMMSON interacts with p32 to increase its mitochondrial targeting in melanoma cells, thereby maintaining the mitochondrial membrane potential and OXPHOS. RPA70 and RPA32 are subunits of the replication protein A complex and are essential for DNA replication [75]. MecciND1 can interact with RPA70 and RPA32, and facilitate them importing into mitochondria. Knockdown of mecciND1 reduces mitochondrial DNA copy numbers, which might affect mitochondrial homeostasis [23].

## 7. Conclusions and Future Perspective

Mitochondria are essential for various biological processes including energy production, metabolism and epigenetic regulation. Although mitochondria are a semi-autonomous organelle, they still need vast nuclear genome-encoded proteins to fulfill their activity and regulate their homeostasis. In addition, some nuclear genome-encoded ncRNAs regulate nuclear genome-encoded mitochondrial protein to mediate mitochondrial homeostasis indirectly. Mitochondrial ncRNAs are “dark matters” and have gradually been revealed to be existent in recent years. They show significant effects on mitochondrial homeostasis because they directly target molecules in mitochondria to regulate gene expression or protein transport (Table 3). However, there are still some problems remaining to be solved. Firstly, a large number of mitochondrial ncRNAs have not been fully characterized. Their function, such as that of the mitochondrial piRNAs, is still unclear. Secondly, the biogenesis process of mitochondrial genome-encoded ncRNAs, including circRNAs and lncRNAs, remains to be illustrated. Thirdly, the functional machineries of mitochondrial miRNAs haven not been totally identified. It is only known that the activities of mitochondrial miRNAs are almost dependent on Ago2. Fourthly, the translocation mechanisms of mitochondrial ncRNAs have not been understood. Some evidence shows the possibility that translocators such as PNPase may be involved the import of mitochondrial ncRNAs. However, this needs further strict verification. The detailed mechanism should be explored. Finally, the strategy for mitochondria-delivering ncRNA mimics or inhibitors should be precisely designed and applied. Delivering the ncRNA mimics or inhibitors to the extra-mitochondrial area in cells might result in a supererogatory effect or phenotype. Utilization of mitochondria-targeting peptides or nanoparticles, such as Zhao et al.’s work [64], is a good choice. In conclusion, mitochondrial ncRNAs can be considered as new regulators of mitochondrial homeostasis, which have significant research and clinical value. 

## Figures and Tables

**Table 1:** The detail of mitochondrial ncRNA profiling.

Methods	NcRNA Type	Sample Source	Data Size	References
Microarray	MiRNA	Mitochondria from adult rat liver	15 identified miRNAs	[9]
Microarray	MiRNA	Mitochondria from the liver of adult male C57BL/6J mice	Patterns of miRNA expression in the mitochondria and liver tissue profiles	[10]
Directional deep sequencing	MiRNA and lncRNA	Mitochondria and mitoplasts from cultured human 143B cells	Full transcriptome	[11]
Microarray	MiRNA	Mitochondria from HeLa cells	57 miRNAs differentially expressed in HeLa mitochondria and cytosol	[12]
RT-qPCR	MiRNAs	Mitochondria from human myoblasts	160 detected miRNA	[13]
RNA-seq	MiRNAs	Mitochondria from HeLa and HEK293 cell	428 and 327 mature miRNAs from HEK293 and HeLa cells	[14]
High-throughput microarray	MiRNAs	Mitochondria from mice heart	78 miRNAs differentially regulated in type 1 diabetic insult and control	[15]
TaqMan^®^ RT-qPCR Array	MiRNAs	Mitochondria from rat hippocampus	285 detected miRNAs	[16]
RNA-seq	MiRNAs	Mitochondria from mice heart	289 detected known miRNAs	[17]
DESeq2	LncRNAs	Mitochondria from HEK293 cells	23 mitochondrial lncRNAs	[18]
RNA-seq	LncRNAs	Mitochondria from HepG2 and HL7702 cells	Transcriptome	[19]
Bioinformatic analysis	PiRNAs	Data from Sripada et al. (PLoS ONE, 2012) and Mercer et al. (Cell, 2011)	29 mitochondrial DNA-derived piRNAs	[20]
Bioinformatic analysis	MiRNAs and piRNAs	Data from Ku et al. (Natl Sci Rev., 2014) and García-López et al. (Biochim Biophys Acta. 2014)	MiRNAs and piRNAs: 56 and 3135 in primordial germ cells, 38 and 4403 in spermatogonia, 43 and 4152 in spermatozoa, 25 and 2703 in oocytes, 38 and 2502 zygotes	[21]
RNA-seq	PiRNAs	Embryonic gonads of mice	The genomic regions, expression level and potential roles of piRNA transcribed mitochondrial DNA	[22]
RNA-seq	CircRNAs	HeLa, HEK293T, RPE-1, HepG2, N2a, and NIH3T3 cells	248 and 268 high-confidence (with ≥2 junction reads) mitochondria-encoded circRNAs in humans and mice	[23]

**Table 2:** Functional ncRNAs identified in mitochondria.

NcRNA	Involved Process	Type	Source	Target	Molecular Effect	Reference
miR-1	OXPHOS	miRNA	C2C12 mice myoblasts	ND1 and COX1 mRNA	Enhancing translation of ND1 and COX1	[34]
miR-21	OXPHOS	miRNA	H9C2 rat cardiomyocytes	CYTB mRNA	Enhancing translation of CYTB	[35]
miR-181c	OXPHOS	miRNA	Neonatal rat ventricular myocytes	COX1 mRNA	Decreasing protein level of COX1	[26]
miR-378	OXPHOS	miRNA	HL-1 human cardiomyocyte	ATP6 mRNA	Decreasing protein level of ATP6	[15,31]
let-7a	OXPHOS	miRNA	MDA-MB-231 and MCF-7 cells	ND4 mRNA	Decreasing protein level of ND4	[36]
miR-2392	OXPHOS	miRNA	CAL-27 and SCC-9 cells	Mitochondria DNA	Enhancing transcription of mitochondrial DNA	[38]
MALAT1	OXPHOS	LncRNA	HepG2 Cells	Mitochondria DNA	Inhibiting methylation of mitochondrial DNA	[19]
GAS5	TCA	LncRNA	MDA-MB-231, MDA-MB-468, HEK293T	MDH2	Promoting the association of FH-MDH2-CS	[18]
LncFAO	β-oxidation	LncRNA	Mice bone marrow-derived macrophages	HADHB	Increasing of HADHB level	[44]
SAMMSON	Mitochondrial translation	LncRNA	Mel501 and SK-MEL-2 cells	P32	Enhancing the mitochondrial localization of P32	[43]
CircPUM1	OXPHOS	CircRNA	Esophageal squamous cell carcinoma cells	UQCRC2	Promoting the association of UQCRC1 and 2	[60]
SCAR	MPTP opening	CircRNA	Primary liver fibroblasts	ATP5B	Inhibiting the interaction between ATP5B and CypD	[64]
CircSmad4	MPTP opening	CircRNA	Mice neonatal cardiomyocytes	VCP	Enhancing the mitochondrial localization of VCP	[59]
MecciND1	Mitochondrial DNA replication	CircRNA	HeLa, HEK293T, RPE-1, HepG2, N2a, and NIH3T3 cells	RPA32/70	Enhancing the mitochondrial localization of RPA32/70	[23]

**Table 3:** Biogenesis, translocation, and molecular function of mitochondrial ncRNAs.

NcRNA	Origination	Biogenesis	Translocation	Molecular Function
MiRNA	Nuclear genome	Transcribed by RNA polymerase II in nucleus. Processed by DROSHA, DGCR8 and Dicer, successively [24].	The pre-miRNAs or mature miRNAs might be imported into mitochondria by Ago2 or PNPase [10,12,14,30,31].	Directly bind mitochondrial mRNAs to promote or inhibit translation [15,26,34,35,36]. Directly bind mitochondrial DNA to inhibit transcription [38]. The function is Ago2-dependent.
	Mitochondrial genome	The existence is not verified yet		
LncRNA	Nuclear genome	Transcribed by RNA polymerase II and processed in nucleus [39].	Might be translocated into mitochondria through their associated mitochondrial proteins [18,19,40].	Directly bind nuclear-derived mitochondrial proteins to affect their translocation, stability, activity or association [18,43,44]. Interact with mitochondrial DNA to regulate DNA methylation [19].
	Mitochondrial genome	Transcribed in mitochondria. Might be processed by mitochondrial tRNA processing factors [50].	Partially being translocated into nucleus [50]. The mechanism is not clear.	Might form RNA-RNA duplexes with their sense mitochondrial mRNAs to regulate stability of sense mRNAs [50].
CircRNA	Nuclear genome	Generated by back-splicing from pre-mRNAs [50,51].	Might be translocated into mitochondria through their associated mitochondrial proteins [59].	Interact with nuclear-derived mitochondrial protein to affect their mitochondrial localization or act as protein scaffold [59,60].
	Mitochondrial genome	The mechanism is not clear. Might be related to the short repeats located at the 5′ and 3′ junction sites [23].	Some distributed in both mitochondria and the cytoplasm [23,63,64]. The mechanism is not clear.	Interact with nuclear-derived mitochondrial protein to affect their mitochondrial localization or protein association [23,64].

## Data Availability

Not applicable.

## References

[1] Gray M.W. (2012). Mitochondrial evolution. Cold Spring Harb. Perspect. Biol..

[2] Tao M., You C.P., Zhao R.R., Liu S.-J., Zhang Z.-H., Zhang C., Liu Y. (2014). Animal mitochondria: Evolution, function, and disease. Curr. Mol. Med..

[3] Garbincius J.F., Elrod J.W. (2022). Mitochondrial calcium exchange in physiology and disease. Physiol. Rev..

[4] Matilainen O., Quirós P.M., Auwerx J. (2017). Mitochondria and Epigenetics—Crosstalk in Homeostasis and Stress. Trends Cell Biol..

[5] Anderson S., Bankier A.T., Barrell B.G., De Bruijn M.H.L., Coulson A.R., Drouin J., Eperon I.C., Nierlich D.P., Roe B.A., Sanger F. (1981). Sequence and organization of the human mitochondrial genome. Nature.

[6] Rath S., Sharma R., Gupta R., Ast T., Chan C., Durham T.J., Goodman R.P., Grabarek Z., Haas M.E., Hung W.H.W. (2021). MitoCarta3.0: An updated mitochondrial proteome now with sub-organelle localization and pathway annotations. Nucleic Acids Res..

[7] Wiedemann N., Pfanner N. (2017). Mitochondrial Machineries for Protein Import and Assembly. Annu. Rev. Biochem..

[8] Virciglio C., Abel Y., Rederstorff M. (2021). Regulatory Non-Coding RNAs: An Overview. Methods Mol. Biol..

[9] Kren B.T., Wong P.Y., Sarver A., Zhang X., Zeng Y., Steer C.J. (2009). MicroRNAs identified in highly purified liver-derived mitochondria may play a role in apoptosis. RNA Biol..

[10] Bian Z., Li L.M., Tang R., Hou D.-X., Chen X., Zhang C.-Y., Zen K. (2010). Identification of mouse liver mitochondria-associated miRNAs and their potential biological functions. Cell Res..

[11] Mercer T.R., Neph S., Dinger M.E., Crawford J., Smith M.A., Shearwood A.-M.J., Haugen E., Bracken C.P., Rackham O., Stamatoyannopoulos J.A. (2011). The human mitochondrial transcriptome. Cell.

[12] Bandiera S., Rüberg S., Girard M., Cagnard N., Hanein S., Chrétien D., Munnich A., Lyonnet S., Henrion-Caude A. (2011). Nuclear outsourcing of RNA interference components to human mitochondria. PLoS ONE.

[13] Barrey E., Saint-Auret G., Bonnamy B., Damas D., Boyer O., Gidrol X. (2011). Pre-microRNA and mature microRNA in human mitochondria. PLoS ONE.

[14] Sripada L., Tomar D., Prajapati P., Singh R., Singh A.K., Singh R. (2012). Systematic analysis of small RNAs associated with human mitochondria by deep sequencing: Detailed analysis of mitochondrial associated miRNA. PLoS ONE.

[15] Jagannathan R., Thapa D., Nichols C.E., Shepherd D.L., Stricker J.C., Croston T.L., Baseler W.A., Lewis S.E., Martinez I., Hollander J.M. (2015). Translational Regulation of the Mitochondrial Genome Following Redistribution of Mitochondrial MicroRNA in the Diabetic Heart. Circ. Cardiovasc. Genet..

[16] Wang W.X., Visavadiya N.P., Pandya J.D., Nelson P.T., Sullivan P.G., Springer J.E. (2015). Mitochondria-associated microRNAs in rat hippocampus following traumatic brain injury. Exp. Neurol..

[17] Wang X., Song C., Zhou X., Han X., Li J., Wang Z., Shang H., Liu Y., Cao H. (2017). Mitochondria Associated MicroRNA Expression Profiling of Heart Failure. Biomed. Res. Int..

[18] Sang L., Ju H.Q., Yang Z., Ge Q., Zhang Z., Liu F., Yang L., Gong H., Shi C., Qu L. (2021). Mitochondrial long non-coding RNA GAS5 tunes TCA metabolism in response to nutrient stress. Nat. Metab..

[19] Zhao Y., Zhou L., Li H., Sun T., Wen X., Li X., Meng Y., Li Y., Liu M., Liu S. (2020). Nuclear-Encoded lncRNA MALAT1 Epigenetically Controls Metabolic Reprogramming in HCC Cells through the Mitophagy Pathway. Mol. Ther. Nucleic Acids.

[20] Kwon C., Tak H., Rho M., Chang H.R., Kim Y.H., Kim K.T., Balch C., Lee E.K., Nam S. (2014). Detection of PIWI and piRNAs in the mitochondria of mammalian cancer cells. Biochem. Biophys. Res. Commun..

[21] Larriba E., Rial E., Del Mazo J. (2018). The landscape of mitochondrial small non-coding RNAs in the PGCs of male mice, spermatogonia, gametes and in zygotes. BMC Genom..

[22] Barreñada O., Larriba E., Fernández-Pérez D., Brieño-Enríquez M.Á., Del Mazo Martínez J. (2022). Unraveling mitochondrial piRNAs in mouse embryonic gonadal cells. Sci. Rep..

[23] Liu X., Wang X., Li J., Hu S., Deng Y., Yin H., Bao X., Zhang Q.C., Wang G., Wang B. (2020). Identification of mecciRNAs and their roles in the mitochondrial entry of proteins. Sci. China Life Sci..

[24] Krol J., Loedige I., Filipowicz W. (2010). The widespread regulation of microRNA biogenesis, function and decay. Nat. Rev. Genet..

[25] Lung B., Zemann A., Madej M.J., Schuelke M., Techritz S., Ruf S., Bock R., Hüttenhofer A. (2006). Identification of small non-coding RNAs from mitochondria and chloroplasts. Nucleic Acids Res..

[26] Das S., Ferlito M., Kent O.A., Fox-Talbot K., Wang R., Liu D., Raghavachari N., Yang Y., Wheelan S.J., Murphy E. (2012). Nuclear miRNA regulates the mitochondrial genome in the heart. Circ. Res..

[27] Gregory R.I., Chendrimada T.P., Cooch N., Shiekhattar R. (2005). Human RISC couples microRNA biogenesis and posttranscriptional gene silencing. Cell.

[28] Chendrimada T.P., Finn K.J., Ji X., Baillat D., Gregory R.I., Liebhaber S.A., Pasquinelli A.E., Shiekhattar R. (2007). MicroRNA silencing through RISC recruitment of eIF. Nature.

[29] Chen H.W., Koehler C.M., Teitell M.A. (2007). Human polynucleotide phosphorylase: Location matters. Trends Cell Biol..

[30] Wang G., Chen H.W., Oktay Y., Zhang J., Allen E.L., Smith G.M., Fan K.C., Hong J.S., French S.W., McCaffery J.M. (2010). PNPASE regulates RNA import into mitochondria. Cell.

[31] Shepherd D.L., Hathaway Q.A., Pinti M.V., Nichols C.E., Durr A.J., Sreekumar S., Hughes K.M., Stine S.M., Martinez I., Hollander J.M. (2017). Exploring the mitochondrial microRNA import pathway through Polynucleotide Phosphorylase (PNPase). J. Mol. Cell Cardiol..

[32] Wang D.D., Shu Z., Lieser S.A., Chen P.L., Lee W.H. (2009). Human mitochondrial SUV3 and polynucleotide phosphorylase form a 330-kDa heteropentamer to cooperatively degrade double-stranded RNA with a 3′-to-5′ directionality. J. Biol. Chem..

[33] Erturk E., Enes Onur O., Akgun O., Tuna G., Yildiz Y., Ari F. (2022). Mitochondrial miRNAs (MitomiRs): Their potential roles in breast and other cancers. Mitochondrion.

[34] Zhang X., Zuo X., Yang B., Li Z., Xue Y., Zhou Y., Huang J., Zhao X., Zhou J., Yan Y. (2014). MicroRNA directly enhances mitochondrial translation during muscle differentiation. Cell.

[35] Li H., Zhang X., Wang F., Zhou L., Yin Z., Fan J., Nie X., Wang P., Fu X.D., Chen C. (2016). MicroRNA-21 Lowers Blood Pressure in Spontaneous Hypertensive Rats by Upregulating Mitochondrial Translation. Circulation.

[36] Harma P., Sharma V., Ahluwalia T.S., Dogra N., Kumar S., Singh S. (2021). Let-7a induces metabolic reprogramming in breast cancer cells via targeting mitochondrial encoded ND. Cancer Cell Int..

[37] Das S., Bedja D., Campbell N., Dunkerly B., Chenna V., Maitra A., Steenbergen C. (2014). miR-181c regulates the mitochondrial genome, bioenergetics, and propensity for heart failure in vivo. PLoS ONE.

[38] Fan S., Tian T., Chen W., Lv X., Lei X., Zhang H., Sun S., Cai L., Pan G., He L. (2019). Mitochondrial miRNA Determines Chemoresistance by Reprogramming Metabolism and Regulating Mitochondrial Transcription. Cancer Res..

[39] Quinn J.J., Chang H.Y. (2016). Unique features of long non-coding RNA biogenesis and function. Nat. Rev. Genet..

[40] Noh J.H., Kim K.M., Abdelmohsen K., Yoon J.-H., Panda A.C., Munk R., Kim J., Curtis J., Moad C.A., Wohler C.M. (2016). HuR and GRSF1 modulate the nuclear export and mitochondrial localization of the lncRNA RMRP. Genes Dev..

[41] Li K., Smagula C.S., Parsons W.J., Richardson J.A., Gonzalez M., Hagler H.K., Williams R.S. (1994). Subcellular partitioning of MRP RNA assessed by ultrastructural and biochemical analysis. J. Cell Biol..

[42] Zhao Y., Liu S., Zhou L., Li X., Meng Y., Li Y., Li L., Jiao B., Bai L., Yu Y. (2019). Aberrant shuttling of long noncoding RNAs during the mitochondria-nuclear crosstalk in hepatocellular carcinoma cells. Am. J. Cancer Res..

[43] Leucci E., Vendramin R., Spinazzi M., Laurette P., Fiers M., Wouters J., Radaelli E., Eyckerman S., Leonelli C., Vanderheyden K. (2016). Melanoma addiction to the long non-coding RNA SAMMSON. Nature.

[44] Nakayama Y., Fujiu K., Yuki R., Oishi Y., Morioka M.S., Isagawa T., Matsuda J., Oshima T., Matsubara T., Sugita J. (2020). A long noncoding RNA regulates inflammation resolution by mouse macrophages through fatty acid oxidation activation. Proc. Natl. Acad. Sci. USA.

[45] Tripathi V., Ellis J.D., Shen Z., Song D.Y., Pan Q., Watt A.T., Freier S.M., Bennett C.F., Sharma A., Bubulya P.A. (2010). The nuclear-retained noncoding RNA MALAT1 regulates alternative splicing by modulating SR splicing factor phosphorylation. Mol. Cell..

[46] Mohammad G., Kowluru R.A. (2021). Nuclear Genome-Encoded Long Noncoding RNAs and Mitochondrial Damage in Diabetic Retinopathy. Cells.

[47] Villegas J., Zárraga A.M., Muller I., Montecinos L., Werner E., Brito M., Meneses A.M., Burzio L.O. (2000). A novel chimeric mitochondrial RNA localized in the nucleus of mouse sperm. DNA Cell Biol..

[48] Villegas J., Burzio V., Villota C., Landerer E., Martinez R., Santander M., Martinez R., Pinto R., Vera M.I., Boccardo E. (2007). Expression of a novel non-coding mitochondrial RNA in human proliferating cells. Nucleic Acids Res..

[49] Landerer E., Villegas J., Burzio V.A., Oliveira L., Villota C., Lopez C., Restovic F., Martinez R., Castillo O., Burzio L.O. (2011). Nuclear localization of the mitochondrial ncRNAs in normal and cancer cells. Cell. Oncol..

[50] Rackham O., Shearwood A.M., Mercer T.R., Davies S.M., Mattick J.S., Filipovska A. (2011). Long noncoding RNAs are generated from the mitochondrial genome and regulated by nuclear-encoded proteins. RNA.

[51] Gao S., Tian X., Chang H., Sun Y., Wu Z., Cheng Z., Dong P., Zhao Q., Ruan J., Bu W. (2018). Two novel lncRNAs discovered in human mitochondrial DNA using PacBio full-length transcriptome data. Mitochondrion.

[52] Kumarswamy R., Bauters C., Volkmann I., Maury F., Fetisch J., Holzmann A., Lemesle G., de Groote P., Pinet F., Thum T. (2014). Circulating long noncoding RNA, LIPCAR, predicts survival in patients with heart failure. Circ. Res..

[53] Ozata D.M., Gainetdinov I., Zoch A., O’Carroll D., Zamore P.D. (2019). PIWI-interacting RNAs: Small RNAs with big functions. Nat. Rev. Genet..

[54] Wang X., Ramat A., Simonelig M., Liu M.F. (2022). Emerging roles and functional mechanisms of PIWI-interacting RNAs. Nat. Rev. Mol. Cell Biol..

[55] Honda S., Kirino Y., Maragkakis M., Alexiou P., Ohtaki A., Murali R., Mourelatos Z., Kirino Y. (2013). Mitochondrial protein BmPAPI modulates the length of mature piRNAs. RNA.

[56] Izumi N., Shoji K., Sakaguchi Y., Honda S., Kirino Y., Suzuki T., Katsuma S., Tomari Y. (2016). Identification and Functional Analysis of the Pre-piRNA 3′ Trimmer in Silkworms. Cell.

[57] Kristensen L.S., Andersen M.S., Stagsted L.V.W., Ebbesen K.K., Hansen T.B., Kjems J. (2019). The biogenesis, biology and characterization of circular RNAs. Nat. Rev. Genet..

[58] Liu C.X., Chen L.L. (2022). Circular RNAs: Characterization, cellular roles, and applications [published correction appears in *Cell*
**2022**, *185*, 2390]. Cell.

[59] Zheng H., Huang S., Wei G., Sun Y., Li C., Si X., Chen Y., Tang Z., Li X., Chen Y. (2022). CircRNA Samd4 induces cardiac repair after myocardial infarction by blocking mitochondria-derived ROS output. Mol. Ther..

[60] Gong W., Xu J., Wang Y., Min Q., Chen X., Zhang W., Chen J., Zhan Q. (2022). Nuclear genome-derived circular RNA circPUM1 localizes in mitochondria and regulates oxidative phosphorylation in esophageal squamous cell carcinoma. Signal Transduct. Target. Ther..

[61] Jeck W.R., Sorrentino J.A., Wang K., Slevin M.K., Burd C.E., Liu J., Marzluff W.F., Sharpless N.E. (2013). Circular RNAs are abundant, conserved, and associated with ALU repeats. RNA.

[62] Salzman J., Chen R.E., Olsen M.N., Wang P.L., Brown P.O. (2013). Cell-type specific features of circular RNA expression. PLoS Genet..

[63] Wu Z., Sun H., Wang C., Liu W., Liu M., Zhu Y., Xu W., Jin H., Li J. (2020). Mitochondrial Genome-Derived circRNA mc-COX2 Functions as an Oncogene in Chronic Lymphocytic Leukemia. Mol. Ther. Nucleic Acids.

[64] Zhao Q., Liu J., Deng H., Ma R., Liao J.-Y., Liang H., Hu J., Li J., Guo Z., Cai J. (2020). Targeting Mitochondria-Located circRNA SCAR Alleviates NASH via Reducing mROS Output. Cell.

[65] Fernandez-Vizarra E., Zeviani M. (2018). Mitochondrial complex III Rieske Fe-S protein processing and assembly. Cell Cycle.

[66] Alavian K.N., Beutner G., Lazrove E., Sacchetti S., Park H.-A., Licznerski P., Li H., Nabili P., Hockensmith K., Graham M. (2014). An uncoupling channel within the c-subunit ring of the F1FO ATP synthase is the mitochondrial permeability transition pore. Proc. Natl. Acad. Sci. USA.

[67] Kwong J.Q., Molkentin J.D. (2015). Physiological and pathological roles of the mitochondrial permeability transition pore in the heart. Cell Metab..

[68] Elrod J.W., Molkentin J.D. (2013). Physiologic functions of cyclophilin D and the mitochondrial permeability transition pore. Circ. J..

[69] Giorgio V., von Stockum S., Antoniel M., Fabbro A., Fogolari F., Forte M., Glick G.D., Petronilli V., Zoratti M., Szabó I. (2013). Dimers of mitochondrial ATP synthase form the permeability transition pore. Proc. Natl. Acad. Sci. USA.

[70] Giorgio V., Burchell V., Schiavone M., Bassot C., Minervini G., Petronilli V., Argenton F., Forte M., Tosatto S., Lippe G. (2017). Ca^2+^ binding to F-ATP synthase β subunit triggers the mitochondrial permeability transition. EMBO Rep..

[71] Ye Y., Shibata Y., Kikkert M., van Voorden S., Wiertz E., Rapoport T.A. (2005). Recruitment of the p97 ATPase and ubiquitin ligases to the site of retrotranslocation at the endoplasmic reticulum membrane. Proc. Natl. Acad. Sci. USA.

[72] Xu S., Peng G., Wang Y., Fang S., Karbowski M. (2011). The AAA-ATPase p97 is essential for outer mitochondrial membrane protein turnover. Mol. Biol. Cell..

[73] Yang X., Zhou Y., Liang H., Meng Y., Liu H., Zhou Y., Huang C., An B., Mao H., Liao Z. (2021). VDAC1 promotes cardiomyocyte autophagy in anoxia/reoxygenation injury via the PINK1/Parkin pathway. Cell Biol. Int..

[74] Wu H., Sun H., Liang X., Lima W.F., Crooke S.T. (2013). Human RNase H1 is associated with protein P32 and is involved in mitochondrial pre-rRNA processing. PLoS ONE.

[75] Liu T., Huang J. (2016). Replication protein A and more: Single-stranded DNA-binding proteins in eukaryotic cells. Acta Biochim. Biophys. Sin..

